# Electrophysiological Properties of Motor Neurons in a Mouse Model of Severe Spinal Muscular Atrophy: *In Vitro* versus *In Vivo* Development

**DOI:** 10.1371/journal.pone.0011696

**Published:** 2010-07-21

**Authors:** Hongmei Zhang, Natallia Robinson, Chiayen Wu, Wenlan Wang, Melissa A. Harrington

**Affiliations:** 1 Department of Biological Sciences, Delaware State University, Dover, Delaware, United States of America; 2 Alfred I. DuPont Hospital for Children, Wilmington, Delaware, United States of America; Sun Yat-Sen University, China

## Abstract

We examined the electrophysiological activity of motor neurons from the mouse model of severe spinal muscular atrophy (SMA) using two different methods: whole cell patch clamp of neurons cultured from day 13 embryos; and multi-electrode recording of ventral horns in spinal cord slices from pups on post-natal days 5 and 6. We used the MED64 multi-electrode array to record electrophysiological activity from motor neurons in slices from the lumbar spinal cord of SMA pups and their unaffected littermates. Recording simultaneously from up to 32 sites across the ventral horn, we observed a significant decrease in the number of active neurons in 5–6 day-old SMA pups compared to littermates. Ventral horn activity in control pups is significantly activated by serotonin and depressed by GABA, while these agents had much less effect on SMA slices. In contrast to the large differences observed in spinal cord, neurons cultured from SMA embryos for up to 21 days showed no significant differences in electrophysiological activity compared to littermates. No differences were observed in membrane potential, frequency of spiking and synaptic activity in cells from SMA embryos compared to controls. In addition, we observed no difference in cell survival between cells from SMA embryos and their unaffected littermates. Our results represent the first report on the electrophysiology of SMN-deficient motor neurons, and suggest that motor neuron development *in vitro* follows a different path than *in vivo* development, a path in which loss of SMN expression has little effect on motor neuron function and survival.

## Introduction

SMA is characterized by motor neuron loss and muscle atrophy, [1], [2], [3], [4] and is caused by homozygous loss or mutation of the survival motor neuron gene 1, SMN1 [5], [6]. A second copy of the gene, SMN2, is almost identical to SMN1 except for several single nucleotide changes, one of which results in alternative splicing and skipping of exon 7 [7], [8] so that 75–90% of SMN transcripts from SMN2 are truncated and are rapidly degraded *in vivo* [9]. While the SMN proteins are ubiquitously expressed, the pathology associated with SMN1 loss appears to be confined to motor neurons [10].

A transgenic mouse considered to be a close model of Type 1 SMA, the most severe form, contains a homozygous deletion of the murine SMN1 gene and carries 2 copies of a human SMN2 transgene [11]. Affected pups appear normal at birth, but within 48 hours show decreased movement, reduced suckling, labored breathing, and small size compared to wild type littermates, and they die before post-natal day 7 (P7). Histological analysis of mutants on post-natal day 1 (P1) shows the normal number of spinal and brainstem motor neurons, but by P3–P5, motor neuron cell bodies in the spinal cord are 20–30% lower in number in the mutants than in age-matched controls, and many of the remaining neurons appear apoptotic. Interestingly, the severity of the SMA phenotype seems outpace the loss of motor neuron cell bodies, as even the most severely affected pups retain 60% or more of spinal motor neurons [11].

In culture, compared to controls, motor neurons from the SMA type 1 mouse show decreased axon length, likely as a results of decreased levels of β-actin [12], [13], as well as reduced clustering of Ca^2+^ channels in their growth cones, and reduced excitability in axon terminals [14]. Imaging studies have also demonstrated denervation of neuromuscular junctions and other synaptic abnormalities that are apparent as early as embryonic day 18.5 [15]. However, to date, there have been no reports of electrophysiological studies examining the excitability of motor neurons from SMN-deficient animal models either in culture or *in vivo*.

Here we report our results measuring the electrophysiological activity of SMN-deficient motor neurons using both single-cell and multi-electrode methods. We used the MED64 multi-electrode array to record extracellularly from motor neurons in the ventral horn of spinal cord slices from 5 and 6 day-old pups from the mouse model for severe SMA. The 64-electrode array allowed us to record simultaneously across the entire ventral horn, giving an overall picture of sharp declines in both the number of active motor neurons and the frequency of spiking activity in SMN-deficient mice compared to their normal littermates. In contrast, whole-cell patch clamp recording of motor neurons cultured from day 13 embryos showed no difference in the electrophysiological activity of cells from SMA embryos compared to littermate controls, even after 21 days in culture. These results represent the first report of the electrophysiological properties of spinal motor neurons from an SMN-deficient model organism, and suggest that motor neuron development *in vitro* follows a developmental path in which motor neuron function and survival is much less dependent on SMN expression than *in vivo* development.

## Methods

Procedures involving animals were conducted in conformity with National Institute of Health Guidelines for the Care and Use of Laboratory Animals. All animal experiments were approved by and conducted in accordance with the Institutional Animal Care and Use Committees of Delaware State University and the Alfred I. duPont Hospital for Children (Animal Welfare Assurance Numbers A3318-01 and A4053-01, respectively).

Mice used for these experiment were of the FVB.Cg-*Smn1^tm1Msd^* Tg(ACTA1-SMN)69Ahmb Tg(SMN2)89Ahmb/J strain and were grown from breeding stock of stock # 008209 obtained from the Jackson Laboratory (Bar Harbor, ME). Two heterozygotes were mated to produce a mixed litter including homozygous mutant pups. Wild type and heterozygous offspring were used as controls as their phenotypes are indistinguishable [16], [17], and we observed no differences in the electrophysiological properties of motor neurons from wild type and heterozygous mice.

### Preparation and recording from spinal cord slices

Mouse pups 5–6 days old were decapitated, and the spinal cords were rapidly removed and immediately placed in ice-cold sucrose artificial cerebrospinal fluid (aCSF) presaturated with 95%O_2_ and 5% CO_2_. The tissue was placed in a shallow groove formed in a gelatin block and glued upright on the stage of a vibratome. After cutting to the lumbar region, transverse spinal cord slices 400 µm thick were cut on a 15° angle in ice-cold sucrose aCSF and then preincubated in Krebs solution oxygenated with 95% O_2_ and 5% CO_2_ at 34°C for at least 1 h before transfer to the recording chamber.

**Figure 1:**
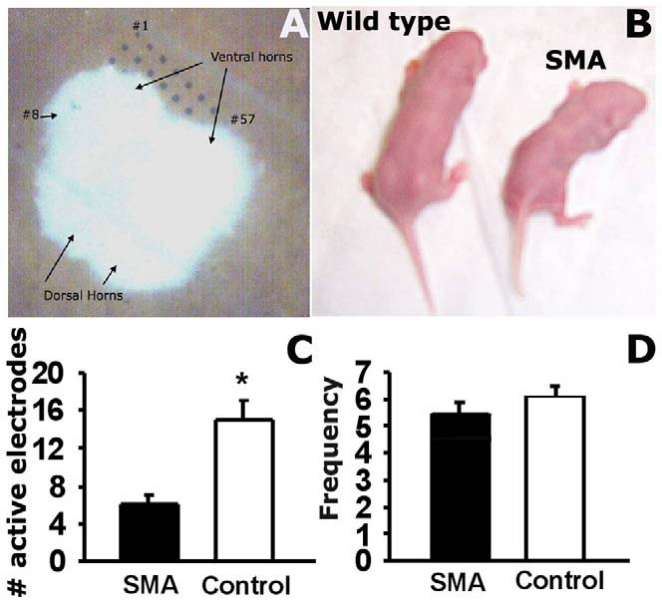
Neural activity is significantly reduced in the spinal cords of SMA mice. **A**. Example of a slice from the lumbar spine with only the ventral horn positioned over the electrodes of the MED probe. **B**. Two littermates from the Smn^−/−^;SMN2 strain of mice at P4 showing the difference in size of the wild type and mutant pups. **C**. Average number of active electrodes recorded for each slice. One Way ANOVA, p<0.0001 **D**. Average spike frequency recorded by each active electrode. *p* = 0.46, One way ANOVA.

Multi-electrode recordings were made with the MED64 system (Automate Scientific) in 50-second increments with a sampling rate of 20 kHz. The MED probe contains 64 electrodes in an 8×8 grid with inter-electrode spacing of 75 µm, and slices were positioned so that only the ventral horn was over the electrodes (covering just over half the grid, see Fig. 1A). Data was filtered in Matlab (The Mathworks Inc., Natick, Massachusetts, USA) by applying a low-pass filter of cut-off 2.5 kHz, and a high-pass filter with cut-off of 85 Hz. The data were detrended with least squares procedures to remove spurious trends [18]. For spike extraction, the standard deviation, δ, was calculated and the voltage threshold was set to ±4.5δ [19]. The spikes were counted and frequency was determined for each 25 sec of recording on active electrodes – defined as those with 50 or more spikes per 50 seconds. To sort the spikes and determine the number of units recorded per electrode, a set of wavelet coefficients was used to extract features in spikes, and a supermagnetic clustering algorithm was used for spike sorting [20]. The significance of the difference between distributions of spike frequencies was tested with the Kruskal-Wallis test, with p-value of less than 0.01 considered to be stationary significant.

### Preparation of primary cultures of motor neurons

Primary cultures were made from the motor neurons of spinal cords from individual day-13 mouse embryos according to methods published earlier [21]. Ventral horn cultures with this method have been shown by immunofluorescence to be almost completely motor neurons [21]. Briefly, 24-well dishes with coverslips were coated over 48 hours with 0.01% poly-L-lysine, then filled with neurobasal medium and kept at 4 °C overnight or until use. The heads of each embryo are collected and stored at -20 °C for genotyping while the ventral horns of each spinal cord were placed individually in 1 ml complete Hibernate E1 (Brainbits, Springfield, IL). The ventral horns were trypsinized in Hank’'s solution for 13 minutes, the trypsin solution was replaced with motor neuron (MN) medium with growth factors and agitated to break up cell clumps. The cell solution was centrifuged at 300 g for 10 minutes at 37°C in MN medium +4% BSA, the supernatant was discarded, and cells resuspended from the pellet with MN medium and counted. Ventral horn neurons from each individual embryo were dispensed to 6 wells. MN medium is 95% Neurobasal medium, 2% B27 (Invitrogen), 2% horse serum, 25 µM β-Mercaptoethanol, 25 µM Glutamic acid, 1% Penicillin-Streptomycin (Mediatech), 50 µM glutamine, 0.01% BDNF (R&D Systems). Cells were incubated 7 days before recording to allow electrical activity to develop.

For genotyping, genomic DNA was extracted from the head (embryos) or tail (pups) and amplified by PCR according to the Jackson Lab protocol.

### Whole-cell patch clamp of cultured neurons

Electrodes were triple pulled from borosilicate glass capillaries and had an impedance of 4–6 MΩ when filled with internal solution containing (mM): K-gluconate, 135; MgCl_2_, 2.0; HEPES, 10.0; EGTA, 0.5; ATP-Mg, 4.0; Na-GTP, 0.5, pH 7.2–7.4, and 290–310 mOsm. QX-314 was added to the intrapipette solution to eliminate action potential when sPSCs were recorded. The extracellular solution contained (mM): NaCl, 150.0; KCl, 3.0; MgCl_2_, 1.0; CaCl_2_, 2.0; glucose, 10.0; and HEPES 10.0, pH 7.2–7.4 with osmolarity of 310–330 mOsm. Motor neurons could be identified by their morphology - a triangular shape with a single well-defined axon and large soma (>20 µm). Recordings of postsynaptic currents began 3 minutes after whole-cell access was established and the current reached a steady state. The input resistance was monitored and the recording as abandoned if it changed more than 15%. Signals were recorded using a MultiClamp700B (Axon Instruments, Foster City, CA) filtered at 1–2 kHz, digitized at 10 kHz, and stored with pCLAMP 10.2 (Axon Instruments). All spontaneous postsynaptic currents (sPSCs) were recorded in voltage-clamp. 20 µM bicuculline, a GABA_A_ receptor antagonist and 10 µM 6-cyano-7-nitroquinoxaline-2,3-dione (CNQX), a specific glutamate non-NMDA antagonist are used to differentiate AMPA receptor- induced or GABA-induced postsynaptic currents. Membrane potential and action potentials were recorded in current-clamp.

### Data analysis

The sPSCs and action potentials were analyzed off-line with a peak detection program (MiniAnalysis, Synaptosoft, Decatur, GA). Measurements of the amplitude and frequency of sPSCs and action potentials were performed over a period of at least 2 min. Events were manually excluded when noise was erroneously identified as sPSCs or action potentials by the program. Background noise levels were typically constant throughout the recording of a single neuron. Significance of results was tested with a one-way ANOVA.

## Results

### Recordings from ventral horns of spinal cord slices

For recordings from spinal cord slices, SMA and control pups (wild type and heterozygous) were identified by body size at day 4 (Fig 1B) and confirmed by genotyping. Spinal cord slices were positioned on the MED64 multi-electrode array so that only the ventral horns were covering the electrodes (Fig 1A). The MED64 was used to record extracellularly from the ventral horn of 11 spinal cord slices from 3 SMA mice and 12 slices from 3 control mice from post-natal day 5 or 6. The recordings demonstrate that the number of spiking motor neurons in the lumbar spinal cord was much lower in SMA mice than control mice. The average number of electrodes recording action potential (spike) activity was only 6±1 per slice in the SMA mice, while in slices from control mice, the number of active electrodes/slice averaged 15±2 (Fig 1C). In the active electrodes, the average spike frequencies in basal conditions were similar in slices from control and SMA mice - 5.48±0.37 Hz in SMA and 6.08±0.42 Hz in control (Fig. 1D). However, spike sorting showed that each electrode recorded fewer neurons in slices from SMA mice. In basal conditions, each active electrode recorded an average of 5 different neurons in slices from control mice, while in slices from SMA mice each active electrode recorded an average of only 4 different neurons.

**Figure 2:**
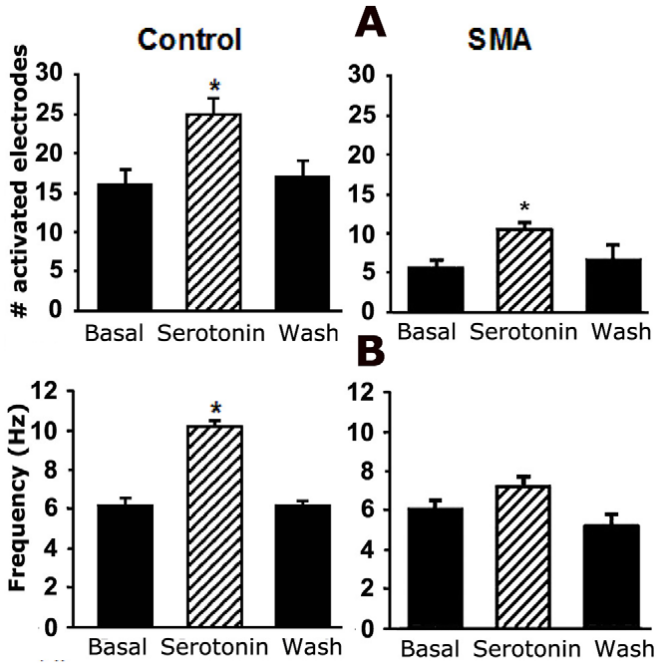
Motor neuron activity in both control and SMA slices is stimulated by serotonin. 50 µM of serotonin was added to the bath and neuronal activity was recorded after 2 minutes. For the wash condition, slices were perfused with aCSF for 10 minutes before recording resumed. **A**. Average number of active electrodes/slice (active electrodes are those that recorded more than 50 spikes in 50 seconds). One way ANOVA, p<0.0001 for both control and SMA. **B**. Average frequency of spikes recorded on active electrodes. One way ANOVA, p<0.0001 for control, p = 0.137 for SMA.

As shown in Figure 2, serotonin significantly increased the number of active spinal motor neurons in both control and SMA mice, increasing both the number of active electrodes per slice from 15 to 25±2 electrodes/slice in controls and from 6 to 11±2 electrodes/slice in SMA (Fig. 2A) while also increasing the number of neurons recorded per active electrode from 5 to 7 in control slices and from 4 to 5 in SMA slices. Reflecting the increase in both the number of active cells recorded by each electrode and the increased spiking activity for those cells, serotonin significantly increased the overall spike frequency on active electrodes in both SMA and control mice although the magnitude of the effect was smaller in SMA mice (6.98±0.49 Hz in SMA slices compared to 10.20±0.27 Hz in controls (Fig. 2B).

**Figure 3:**
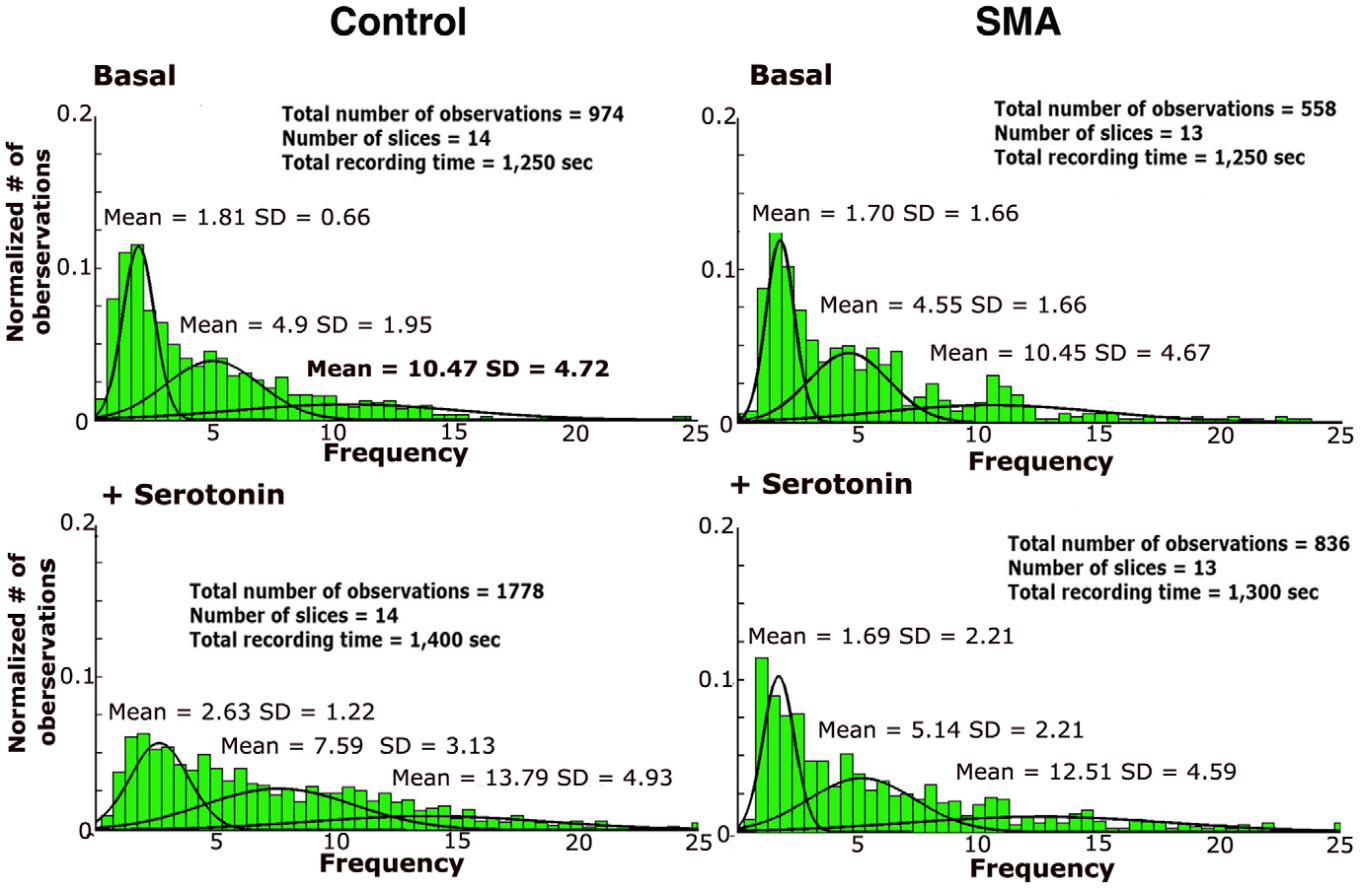
In SMA slices the overall distribution of spike frequencies is not significantly changed by serotonin. Histograms of spike frequency comparing slices from control and SMA mice. The number of observations was normalized to the total number of observations in each slice and divided into. 0.5 Hz bins. The distributions were fitted with mixed Gaussian approximations using the Expectation-Maximization (EM) algorithm [26]. Serotonin significantly shift the frequency distribution in control slices (p<0.0001), but the shift is not significant in the SMA slices (p = 0.1433; Kruskal-Wallis).

While serotonin does cause a significant increase in the number of active neurons and the average spike frequency in slices from SMA mice as well as control mice, an examination of the distribution of spike frequencies on the active electrodes shows some differences between the SMA mice and the control. Frequency distributions for both control and SMA slices could be fit with three Gaussian peaks, but in control slices serotonin caused a much larger change in the frequency distribution, dramatically shifting activity from the low frequency range into the middle and high frequency peaks (Fig. 3). In contrast, in the SMA slices, serotonin had almost no effect on the low frequency peak, only shifting the middle and high range peaks to slightly higher frequencies. Overall, there was a significant change in the distribution of the frequencies between basal and serotonin for the control slices, however, the difference in the distributions was not significant for the SMA slices (Fig 3).

**Figure 4:**
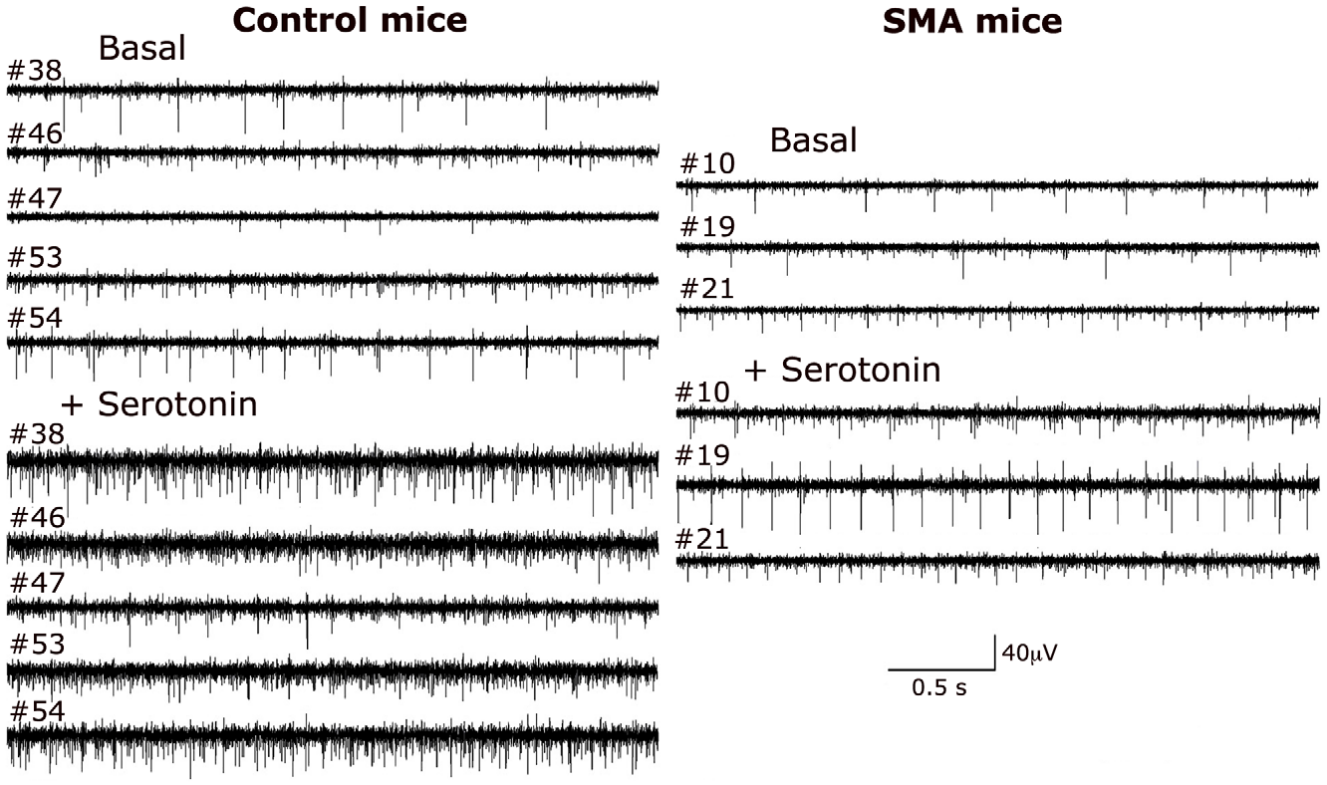
Sample traces show the reduced activity recorded from the ventral horns of SMA mice. Sample traces showing spike activity recorded from all active electrodes in a single slice from a control and an SMA mouse showing activity in both basal and serotonin-stimulated conditions. Slices from SMA mice had many fewer active electrodes than controls, and showed fewer neurons active after serotonin treatment.

Examples of spike activity recorded simultaneously from multiple electrodes in slices from control and SMA mice are shown in figure 4. The heterogeneity of spike types in control slices after serotonin illustrates how the drug increases spike frequency by recruiting additional cells to be active, while sample traces from SMA slices show less size variation in spike activity indicating fewer active neurons recorded by each electrode.

In addition to serotonin, three other pharmacological agents were tested for their effect on the activity of motor neurons recorded from ventral horn slices. Three quantities were calculated and are shown in Table 1: the average number of electrodes recording activity; the average frequency of spikes on each electrode, and the average number of spikes per minute recorded from each slice. This final quantity sums the spikes recorded on all electrodes recoding neural activity. Of the compounds tested, only GABA (20 µM) had a significant effect on slice activity, reducing the total number of spikes per slice, but only in the control slices. None of the compounds caused a significant change in slice activity in SMA slices (Table 1).

**Table 1:** Pharmacological agents cause little change in activity of control or SMA slices.

Condition	Control			SMA		
	Average # Active electrodes	Spike Frequency	Average # spikes/slice	Average # Active electrodes	Spike Frequency	Average # spikes/slice
Control	14±1	6.08±0.34	5018±901	6±1	5.65±0.38	2030±598
Acetylcholine	14±1	5.88±0.35	5075±880	6±1	5.39±0.32	1787±448
Wash	13±1	6.02±0.37	4804±910	6±1	5.83±0.49	2088±533
Control	16±2	6.27±0.31	6008±690	7±1	5.43±0.47	2292±357
Bicuculline	16±2	5.96±0.39	5645±913	7±1	6.18±1.22	2604±362
Wash	16±2	5.67±0.46	5515±946	7±1	5.63±0.69	2319±492
Control	16±1	6.37±0.39	6059±734	6±1	5.62±0.66	2029±667
GABA	14±1	5.74±0.45	4824±790*	6±1	5.14±0.41	1832±600
Wash	15±1	6.70±0.49	6023±873	6±1	5.74±0.61	2075±463

Acetylcholine (100 µM), bicuculline (10 µM) or GABA (20 µM) were bath applied to the slice and recording was resumed after 2 minutes. For wash, slices were perfused with aCSF for 10 minutes before recording was resumed. Averages are calculated for 12 slices from 3 control mice and 11 slices from 3 SMA mice.

*p<0.05 (One-way ANOVA). Active electrodes are those electrodes recording spiking activity of at least 1 Hz over a 50 second period. Number of spikes per minute totals the number of spikes recorded on all electrodes recording neural activity from a slice.

### Whole-cell recordings from primary motor neuron cultures

**Figure 5:**
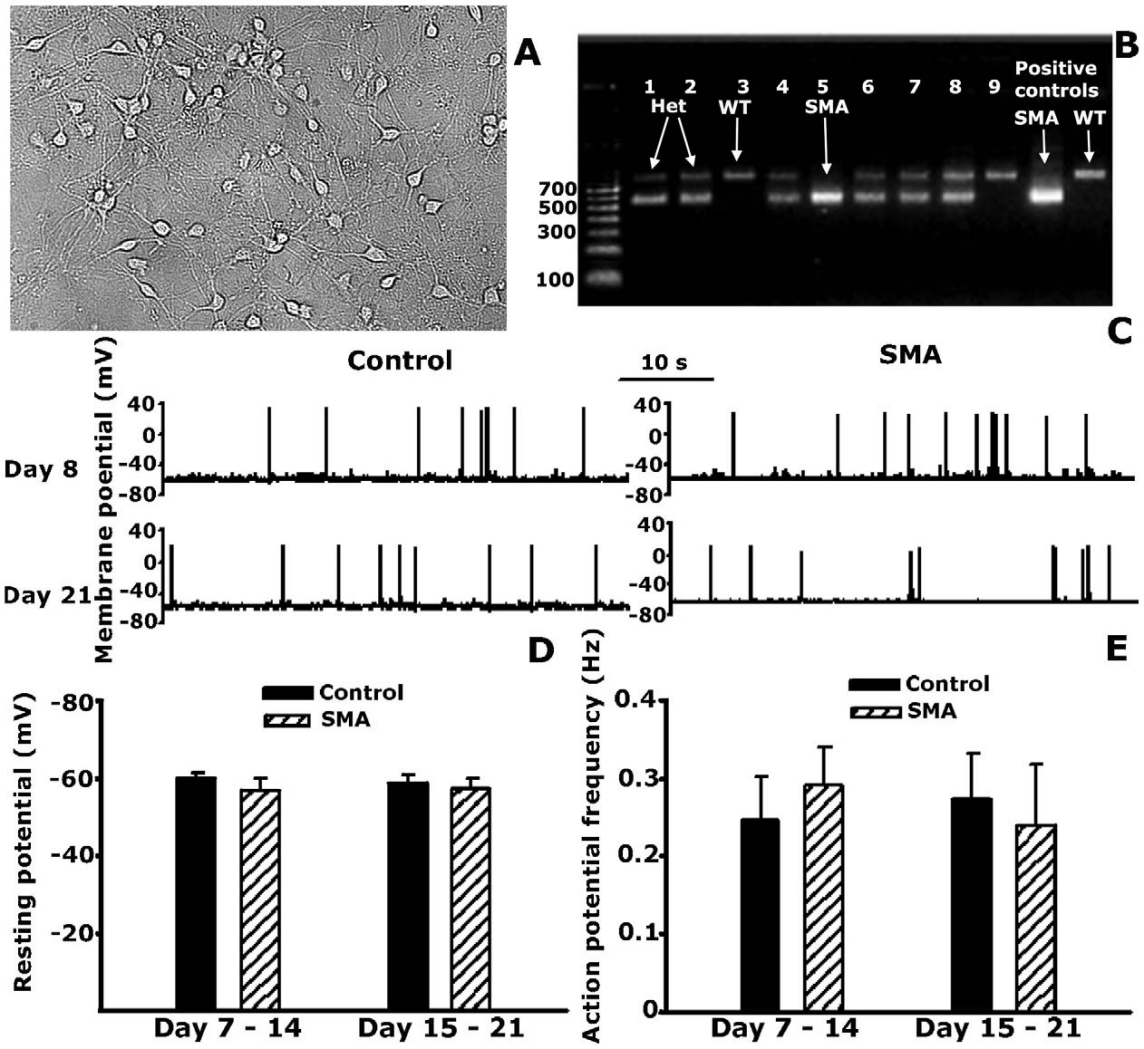
No difference in spiking activity in motor neurons cultured from wild type and SMA embryos. **A**. Sample of cells from a ventral horn culture from an E13 embryo (wild type, 7 days in culture) showing the large somas and extensive network of cell processes. **B**. Sample genotyping result using the protocol from the Jackson Lab. Wild type band is 800 bp. Band from SMN1 knock-out is 500 bp. **C**. Sample traces showing the membrane potential and action potentials from control and SMA mice. **D**. Summary data of the resting membrane potential comparing the 2^nd^ and 3^rd^ week culture in control and SMA mice. **E**. Summary data of the frequency of action potential comparing the 2^nd^ week and 3^rd^ week culture in control and SMA mice. We did not find any significant difference between the average membrane potential or action potential frequency between the control and SMA cultures (*p*>0.05, One-way ANOVA).

In culture, neurons dissociated from ventral horns of wild type and SMA mice form extensive processes, and are indistinguishable in appearance. Whole-cell recordings were made from neurons cultured from 4 separate SMA embryos and 5 wild-type embryos. Action potential activity could be recorded from the cells starting on culture day 6. Patch-clamp recordings with cultured motor neurons were conducted between culture days 7–21 (Fig. 5,B,C). Initially the data was pooled per day of culture, but as no differences between the SMA and wild-type cells could be observed, the results were pooled into two groups: recordings made between culture day 7 to day 14 which correspond to the time that pups are born and SMA pups typically are alive; and recordings made between culture days 15–21, which correspond to the second post-natal week beyond the time that SMA pups survive.

The resting membrane potential (RMP) recorded between day 7 to 14 averaged −59.8±1.2 mV in 13 control cells and −56.7±3.2 mV in 11 SMA cells. Between days 15–21 the average RMP was – 58.3±2.4 mV in 12 control cells and −57.2±2.5 mV in 13 SMA cells (Fig. 5D). There is no significant difference between the control and SMA groups regardless of the recording period (Fig. 5; *P*>0.05, One way ANOVA). Similarly, action potential frequency from day 7–14 averaged 0.24±0.06 Hz in 13 control cells and 0.29±0.05 Hz in 11 SMA cells, while from day 15–21 the action potential frequency averaged 0.27±0.06 Hz in 12 control cells and 0.23±0.08 Hz in 13 SMA cells (Fig. 5E). There is no significant different between cells in the control and SMA cultures (Fig. 4; *P*>0.05, One way ANOVA). In addition to the lack of difference in the electrical activity and resting membrane potential between SMA and wild type cells, no difference was observed in cell survival as long as day 21 of culture.

**Figure 6:**
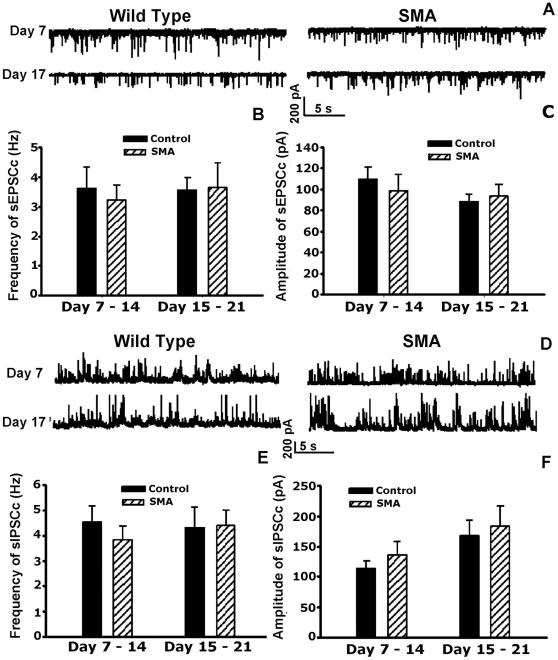
Excitatory and inhibitory input in motor neurons cultured from wild type and SMA mice. **A**. Sample traces of the sEPSCs recorded on day 7 and day 17 in control and SMA cells. **B**. Summary data of the frequency of sEPSCs during the 2^nd^ and 3^rd^ week of culture in control and SMA cells. **C**. Summary data of the amplitude of sEPSCs during the 2^nd^ and 3^rd^ week culture in control and SMA cells. **D**. Sample traces of the sIPSCs at day7 and day17 in control and SMA mice. **E**. Summary data of the frequency of sIPSCs during the 2^nd^ and 3^rd^ week of culture in control and SMA cells. **F**. Summary data of the amplitude of sIPSCs during the 2^nd^ and 3^rd^ week culture in control and SMA mice. We did not find any significant difference in the frequency or amplitude of either excitatory or inhibitory PSPs between the control and SMA cultures (*p*>0.05, One-way ANOVA).

Spontaneous excitatory post-synaptic potentials (EPSCs) were recorded in the presence of bicuculline (20 µM) and strychnine (2 µM) to block inhibitory currents, and sample traces are shown in Fig 6A. From day 7–14, the frequency of sEPSCs was 3.6±0.7 Hz in 9 control cells and 3.2±0.5 Hz in 9 SMA cells, while from day 15–21, the frequency of sEPSCs averaged 3.6±0.4 Hz in 9 control cells and 3.6±0.8 mV in 9 SMA cells (Fig. 6B). In addition, the average amplitude of sEPSCs recorded from day 7–14 did not differ significantly between control and SMA cells (108±12 pA for 9 control and 98±15 pA for 9 SMA cells). Similarly, from day 15–21, there was no significant different in the amplitude of sEPSCs between control and SMA cells (88±7 pA in 9 control cells and 93±11 pA in 9 SMA cells; Fig. 6C).

Spontaneous inhibitory post-synaptic potentials (IPSCs) are recorded in the presence of 10 µM CNQX to block excitatory currents, and sample traces are shown in Fig. 6D. As with the sEPSCs, there was little difference between wild type and SMA cells. The sIPSC frequency recorded between days 7–14 was 4.6±0.6 Hz for 10 in control cells and 3.8±0.5 Hz in 13 SMA cells, and from day 15–21 was 4.3±0.8 Hz in 9 control cells and 4.4±0.6 Hz in 10 SMA cells (Fig. 6E). Similarly, sIPSC amplitude showed no significant change between wild type and SMA cells averaging 113±12 pA in 10 control cells and 135±22 pA in 13 SMA cells from day 7–14 and 168±25 pA in 9 control cells and 183±33 pA in 9 SMA cells between day 15–21 (Fig. 6F).

## Discussion

This paper presents results with a mouse model for type 1 SMA that represent the first report of the electrophysiological properties of SMN-deficient motor neurons. Multi-electrode recordings of ventral horns from mice modeling the most severe form of SMA record many fewer active neurons on P5 and P6 than control littermates. The decrease in electrically active cells is observed both in the smaller number of sites in the ventral horns where neural activity can be recorded, and in the smaller number of spiking neurons recorded by each electrode. Of the pharmacological agents tested, serotonin was the only one that had much effect on the activity of the ventral horn neurons. In both control and SMA slices, serotonin recruited more neurons to be active by increasing both the number of electrodes recording activity and the average number of neurons recorded by each electrode. Serotonin also increased the spike frequency of the active cells. However, on the whole, the magnitude of the stimulating effects of serotonin was lower in SMA slices compared to controls. The total distribution of spike frequencies showed that in control slices, serotonin shifted all peaks of the distribution toward higher frequencies, and the effect was most pronounced at lower spike frequencies. In contrast, in the SMA slices, there was little change in the mean and the proportion of observations in the lowest frequency peak, and only modest shifts in the means of the medium and higher frequency peaks. Overall, our slice recordings from the SMA model mice suggest that by P5–P6 many motor neurons in the ventral spinal cord are no longer active, and those that remain are less active overall and less able to be stimulated by modulating agents. These results are consistent with histological studies with this mouse model which showed the normal number of spinal and brainstem motor neurons at PI, but a decrease in motor neuron cell bodies in the spinal cord of 20–30% by P3–P5 compared to age-matched controls [11]. These results are also consistent with electrophysiological studies of human SMA subjects, which found decreases in motor unit number associated with symptom onset and progressing over time [22].

In contrast, when SMA motor neurons are removed from the spinal cord of day 13 embryos and cultured *in vitro*, no differences could be observed between their electrophysiological properties and those of neurons from control littermates. Similar to an earlier report [12], cell survival appeared to be the same up to culture day 21 which is approximately equivalent to post-natal day 14. Not only did the SMA cells survive, there was no difference in their resting membrane potential or the frequency of spontaneous action potential activity compared to wild type motor neurons. In addition, no difference was observed in synaptic function between the wild type and SMA neurons in culture, in spite of several studies with mutant pups showing reduced excitability in the axon terminals, impaired synaptic vesicle release and neurotransmission failures at neuromuscular synapses [16], [23], [24]. Recordings of the frequency and amplitude of spontaneous excitatory post-synaptic potentials (many of which are likely to represent synapses between motor neurons) found no difference between wild type and SMA neurons even after 21 days in culture. Inhibitory post-synaptic potentials were also the same in mutant and control neurons, indicating that cultured wild type and mutant neurons were equally responsive to GABA, while in slice recordings, only control cells were inhibited by GABA. These results suggest that there is no difference in the number and efficacy of synapses formed by networks of wild type and SMN-deficient motor neurons grown in culture.

Taken together, these results suggest that motor neuron development in culture proceeds down a different developmental pathway than *in vivo* development in the spinal cord, and that *in vitro* development leads to motor neurons that are much less dependent on expression of SMN for survival and function. The lack of dependence of cultured motor neurons on SMN expression indicates that care must be taken in using motor neuron culture as a model system for studying SMA, but also suggests that a deeper understanding of how motor neuron development in culture differs from development *in vivo* may provide key insights into the pathophysiology of SMA.

One major difference between *in vitro* and *in vivo* development is contact with muscle cells. For primary culture, motor neurons are harvested from E13 embryos, a stage when the motor neuron axons have begun to leave the spinal cord, but have not yet made synapses on muscle cells [25]. It is possible that contact with muscle cells starts motor neurons down a developmental path in which SMN expression is critical for cell function and survival. In the absence of that contact, motor neurons may be no more dependent on SMN expression than other types of neurons.
